# Elaborate Tongue Fasciculations Going Down to the Neck: A Rare Case of Sporadic, Young-onset Amyotrophic Lateral Sclerosis with Bulbar Symptoms, from Pakistan

**DOI:** 10.7759/cureus.4600

**Published:** 2019-05-05

**Authors:** Khushboo Nusrat, Samar Mahmood, Shayan Marsia, Khalid Mahmood

**Affiliations:** 1 Internal Medicine, Dow University of Health Sciences (DUHS), Karachi, PAK

**Keywords:** amyotrophic lateral sclerosis, tongue fasciculations, bulbar onset als, neurodegenerative disease, motor neuron diseases, motor examination, riluzole, benzothiazine

## Abstract

Amyotrophic lateral sclerosis (ALS) is a rare, progressive neurodegenerative disease, part of the spectrum of motor neuron diseases. This disease is divided on the bases of heritability, with majority of the cases being sporadic and phenotype, with eight recognized patterns-each with its respective symptoms, rate of progression, and prognosis. Here, we report a case of sporadic, bulbar-onset ALS, unique in its presentation as our patient had fully progressed bulbar symptoms, at the age of 28 years-where other cases of bulbar ALS are associated with much older ages and have a predisposition for the female gender. His prominent and elaborate tongue fasciculations going all the way down to the neck and rendering him incapable of holding his tongue out made for an additional reason of our special interest in the case and the keenness to report it.

## Introduction

Amyotrophic lateral sclerosis (ALS) is a progressive neurodegenerative disease, part of the spectrum of motor neuron diseases. Upper motor neuron and lower motor neuron (LMN) are involved at the bulbar and spinal levels, thus leading to a presentation which can be localized to either one or both these regions [1]. Though variations in the incidence of ALS have been reported based on geography, the worldwide incidence is found to be 1.79/100,000 in males and 1.45/100,000 in females [2]. Despite the rarity of the condition, it has serious financial and social implications on sufferers, making it essential to research and understand the condition thoroughly [3]. Only 5%-10% of cases are familial, with the remaining 90% of documented cases being sporadic [4].

Furthermore, the peak age of onset for sporadic cases is 58-63 years while peak age of onset for familial cases is 47-52 years [5]. Other than the division based on heritability, ALS can also be divided based on how it presents, with 25% patients presenting with bulbar symptoms, and the remaining patients presenting with limb involvement [6]. Additionally, young-onset ALS has also been noted where, patients are presenting with similar symptoms but before the age of 45 and these patients are thought to have the least incidence of bulbar-onset ALS [7].

We report a case of sporadic bulbar-onset ALS, unique in its presentation as our patient had fully progressed bulbar symptoms, at the age of 28 years. His prominent and elaborate tongue fasciculations going all the way down to the neck and rendering him incapable of holding his tongue out made for an additional reason of our special interest in the case and the keenness to report it.

## Case presentation

A 28-year-old male patient presented to the outpatient department with a 10-month history of difficulty in chewing and swallowing food, which was worse for liquids initially but progressed to ingestion of solids as well. In addition, the patient complained of an inability to communicate effectively, which had progressed over the course of his disease. He experienced excessive salivation and had noticed marked weight loss, although undocumented. Furthermore, he complained of involuntary persistent twitching, most notable over the chin, tongue, upper and lower limbs. According to the patient, the difficulty in chewing and swallowing developed prior to the muscle twitching. On further inquiry, he had not experienced mental deterioration and there was no history of trauma, fever, or fits. His family history was negative for a similar illness, there was no consanguinity of parents, and he had no history of working with chemicals which are known to herald the onset of such symptoms.

On neurological examination, the patient had slurred speech with a nasal tone, marked tongue atrophy and severe tongue fasciculations, which were seen on the chin and neck as well (Video 1). His tongue muscles proved to be extremely weak as there was an inability to keep his tongue protruded even for a few seconds, along with an inability to push his tongue against the underside of his cheeks. His cough reflex was weak, and the sternocleidomastoid muscle was visibly wasted. On motor examination of the upper limbs, we noted fasciculations on the forearms, but bulk, tone, and power seemed to be intact. On motor examination of the lower limbs, we noted fasciculations on the legs. Power in both lower limbs was 3/5 and on eliciting deep tendon reflexes, they proved to be brisk. Babinski’s sign was positive as well. Mental and sensory examinations were normal.

**Video 1:** Severe tongue fasciculations going down to the chin and neck (bulbar-onset ALS). ALS, amyotrophic lateral sclerosis.

The relevant investigations were carried out to rule out our differentials which consisted of an infectious etiology, neurological tumor, Kennedy’s disease, Fredrich’s ataxia, and spinocerebellar degeneration. Electromyography showed decreased motor units with evident denervation changes. Nerve conduction studies were normal. Imaging was carried out as well, but the MRI scans showed no significant lesions, thus ruling out space-occupying or degenerative lesions of any kind. Viral and bacterial tests were also negative. The patient was counseled regarding the severity of his condition and was prescribed benzothiazine, along with being offered speech therapy. However, his symptoms progressed rapidly and five months after the diagnosis, he developed respiratory failure and had to be put on ventilatory support. The patient passed away a month later.

## Discussion

Through research, eight phenotypes of ALS have been recognized: classic, bulbar, flail arm, flail leg, pyramidal, respiratory, pure upper motor neuron, and pure LMN. Based on a review of the literature, it is seen that there are distinctions between each clinical phenotype-which may or may not be evident-regarding the onset of symptoms, rate of progression, and prognosis. The review identified the classic ALS phenotype as being the most common one, normally characterized by symptoms in the upper and motor limbs, combined with pyramidal signs in most patients. Interestingly, the review also mentioned the bulbar variant of the disease as being the least common in young patients, with most of the patients affected being above 80 years. Furthermore, it also established that less than 10% of patients who suffered from the bulbar phenotype were less than 39 years. This is in stark difference from our case, where the patient presented with clear and progressive bulbar symptoms and signs, before the age of 30 years. Other than that, a clear preponderance of the bulbar phenotype has been witnessed in females-again, making our case distinct in its presentation in a male [8].

The presentations seen in ALS patients vary according to the phenotype involved. Bulbar-onset ALS is normally associated with symptoms which involve the swallowing and speech mechanisms of an individual. Patients experience difficulty in swallowing, which is worse for liquids compared to solids. Dysarthria and dysphagia are also part of the presentation, often accompanied by tongue weakness, atrophy, and fasciculations. Additionally, bulbar-onset ALS patients may also present with cognitive changes, often leading to personality and behavioral changes which are initially noted by those around them. Disease progression can ultimately lead to respiratory failure and death. Though the bulbar phenotype of ALS presents with the signs and symptoms detailed above, they can also be seen in the other clinical variants [9]. Studies have shown that the bulbar-onset ALS has the worse prognosis, along with the respiratory phenotype, with death occurring within two years. This signifies the rate at which the disease progresses, which is in conjunction with the disease progression of our patient as well-who developed severe fasciculations which began from the tongue and went down to the neck. His limbs also displayed severe twitching and unfortunately, five months after the diagnosis was made, our patient developed respiratory failure and was put on ventilatory survival [8].

Owing to the differences in presentation seen in our case, compared to most bulbar-onset ALS patients, we identified our main differential diagnoses as: a neurological tumor localized to the brainstem, Kennedy’s disease, Fredrich’s ataxia, and spinocerebellar degeneration. The first differential was ruled out through imaging modalities such as magnetic resonance imaging. Kennedy’s disease or spinobulbar muscular atrophy is an X-linked disorder which presents in men over the age of 40 years. Patients suffering from the condition display a progressive LMN condition which can affect the bulbar region and lower limbs, with fasciculations over the chin being a significant clue towards diagnoses. However, the progression of disease is slow, and sufferers can live a normal life with the disease being ruled out through a negative genetic test [10]. Fredrich’s ataxia was also part of our differential based on the bulbar symptoms in our patient, but this condition also demonstrates decreased or absent reflexes in the lower limb and abnormalities of the peripheral sensory system, the latter being completely absent in our patient, along with a diagnostic genetic test [11]. Inflammatory conditions were also part of our differential diagnoses but were ruled out based on the relevant investigations. The diagnosis of ALS was made once the other possible diagnoses were ruled out, based on the El Escorial revised criteria [12].

As ALS is thought to occur due to glutamate levels within the body increasing to toxic levels and leading to destruction of neurons, Riluzole has become the mainstay of medical management, owing to its ability to block the release of glutamate [13]. Through a review of the literature, it has been established that Riluzole can be considered a disease-modifying drug for ALS, as there is a median increase in survival of two-three months, with no serious side-effects, making it the drug of choice for this condition [14]. However, the severity of the condition calls for a multidisciplinary approach towards management, where the patient often requires assistance from the physiotherapists, occupational therapists, speech therapists, and pulmonary physicians [15]. However, the bulbar phenotype of ALS has the worst prognosis, owing to which patients are unable to survive for a period greater than two years, as was evident in our patient [8].

## Conclusions

Thus, we see a rare case of rapidly progressive, bulbar-onset ALS, in a male patient aged 28 years, with associated, severe tongue fasciculations. This report presents the case in light of the detailed associated history, relevant clinical examinations, devised differentials, and necessary investigations to streamline the diagnosis. It further discusses the progression of the various types of the disease as well as approaches of management, depicting a holistic picture of the disease.
